# Physicians’ Perspectives of Telemedicine During the COVID-19 Pandemic in China: Qualitative Survey Study

**DOI:** 10.2196/26463

**Published:** 2021-06-01

**Authors:** Jialin Liu, Siru Liu, Tao Zheng, Yongdong Bi

**Affiliations:** 1 Department of Medical Informatics West China Hospital Sichuan University Chengdu China; 2 Department of Otolaryngology West China Hospital Sichuan University Chengdu China; 3 Department of Biomedical Informatics University of Utah Salt Lake City, UT United States

**Keywords:** telemedicine, COVID-19, survey, physician

## Abstract

**Background:**

Generalized restriction of movement due to the COVID-19 pandemic, together with unprecedented pressure on the health system, has disrupted routine care for non–COVID-19 patients. Telemedicine should be vigorously promoted to reduce the risk of infections and to offer medical assistance to restricted patients.

**Objective:**

The purpose of this study was to understand physicians’ attitudes toward and perspectives of telemedicine during and after the COVID-19 pandemic, in order to provide support for better implementation of telemedicine.

**Methods:**

We surveyed all physicians (N=148), from October 17 to 25, 2020, who attended the clinical informatics PhD program at West China Medical School, Sichuan University, China. The physicians came from 57 hospitals in 16 provinces (ie, municipalities) across China, 54 of which are 3A-level hospitals, two are 3B-level hospitals, and one is a 2A-level hospital.

**Results:**

Among 148 physicians, a survey response rate of 87.2% (129/148) was attained. The average age of the respondents was 35.6 (SD 3.9) years (range 23-48 years) and 67 out of 129 respondents (51.9%) were female. The respondents come from 37 clinical specialties in 55 hospitals located in 14 provinces (ie, municipalities) across Eastern, Central, and Western China. A total of 94.6% (122/129) of respondents’ hospitals had adopted a telemedicine system; however, 34.1% (44/129) of the physicians had never used a telemedicine system and only 9.3% (12/129) used one frequently (≥1 time/week). A total of 91.5% (118/129) and 88.4% (114/129) of physicians were willing to use telemedicine during and after the COVID-19 pandemic, respectively. Physicians considered the inability to examine patients in person to be the biggest concern (101/129, 78.3%) and the biggest barrier (76/129, 58.9%) to implementing telemedicine.

**Conclusions:**

Telemedicine is not yet universally available for all health care needs and has not been used frequently by physicians in this study. However, the willingness of physicians to use telemedicine was high. Telemedicine still has many problems to overcome.

## Introduction

The COVID-19 pandemic has drastically impacted global health care and dramatically changed the practice of health care [1,2]. Pervasive movement restriction and the unprecedented pressure on the health system has disrupted routine care for non–COVID-19 patients. Therefore, the COVID-19 pandemic has rapidly and fundamentally altered the pattern medical practitioners follow to provide care to patients. To better mitigate and manage the spread of COVID-19, hospitals can replace some routine medical services with telemedicine to improve the efficiency of their health care system [3].

Since telemedicine was first introduced in the late 1950s, it has been used in all aspects of health care with the widespread use of telecommunication technology [4]. In a bibliometric analysis of health technology and informatics, telemedicine was identified as one of the three most common keywords [5]. Now the application of telemedicine has expanded from providing health care services in hospitals, outpatient departments, and specialist offices, as well as between health care providers, to deliver care in patients’ homes [6]. One study has shown that achieving instant patient access, overcoming service gaps, and improving quality are important motivators for physicians to implement telemedicine in acute care units, while issues such as licensure, credentialing, malpractice protection, cost, and reimbursement are barriers to successful implementation [7]. Another study identified that the main challenges in establishing telemedicine systems in developing countries are the high cost of telemedicine systems and solutions, slow clinical acceptance of telemedicine and resistance to change, and lack of the required information and telecommunications technology infrastructure for telemedicine. The major recommendations include setting clear goals for the project, selecting the appropriate application of medical areas and priorities, and adopting user-friendly interfaces [8].

Our study focused on the context of COVID-19 to investigate the current usage of telemedicine during the pandemic in China. With the development of telemedicine, the evaluation of telemedicine is particularly important [9]. The selection of statistical methods is a key step in telemedicine evaluation. The following statistical methods have been used extensively in telemedicine evaluation: statistical comparison, agreement evaluation (κ statistic), and the receiver operating characteristic curve [10-14]. Since telemedicine evaluation needs to explore various outcomes, it may be appropriate to evaluate from a multidisciplinary perspective and use various statistical methods [10]. However, there is a lack of empirical research about telemedicine in different specialties [15]. Some researchers have provided theoretical and practical evidence on the significance of using telemedicine and virtual care to treat patients remotely during the COVID-19 pandemic [16]. Major health organizations around the world, including the World Health Organization, the US Centers for Disease Control and Prevention, and the American Medical Association, have advocated for the use of telemedicine during the COVID-19 pandemic and have taken steps to promote its use [17-19]. During the COVID-19 pandemic, telemedicine has been considered a useful tool to relieve pressure on overburdened health systems. Physicians’ willingness or unwillingness to use telemedicine is a well-known factor in facilitating or inhibiting telemedicine acceptance [20]. In addition, some studies noted that the adoption of telemedicine systems depends on physicians’ and patients’ satisfaction with the use of the telemedicine service [21]. However, physicians’ perspectives on telemedicine visits have not been fully investigated.

To promote the usage of telemedicine during the COVID-19 pandemic, the current state of telemedicine and physicians’ perspectives need to be explored. To better understand the development of telemedicine during the COVID-19 pandemic and to summarize the problems of telemedicine in response to the pandemic, we collected the opinions and suggestions of 148 young and middle-aged physicians regarding the application of telemedicine during the COVID-19 pandemic. These recommendations provide valuable insights for developing and improving telemedicine in the later stages of the COVID-19 pandemic and play an important role in guiding the development of telemedicine.

## Methods

### Participants

We surveyed all physicians (N=148), from October 17 to 25, 2020, who attended the clinical informatics PhD program at West China Medical School, Sichuan University, China. These physicians passed the program’s application and examination process and the hospital academic committee’s review. They had high levels of informatics literacy and a certain understanding of information technology and telemedicine at their hospitals. The physicians came from 57 hospitals in 16 provinces (ie, municipalities) across China, 54 of which are 3A-level hospitals, two are 3B-level hospitals, and one is a 2A-level hospital. The Ministry of Health in China categorizes Chinese hospitals into three levels—primary, secondary, and tertiary hospitals—based on the quality of the health care provided, medical education, and research. Each level is further subdivided into three subsidiary levels: A, B, and C. In 2019, there were 1246 hospitals at the 3A level [22], the highest level of hospitals in China.

This study was approved by the Institutional Review Board at West China Medical School, Sichuan University (IRB17-75).

### Procedure

We conducted a survey using semistructured and open-ended questions to understand physicians’ perspectives of telemedicine during the COVID-19 pandemic in China. Prior to completing the survey, the physicians spent more than 3 hours on coursework related to telemedicine. The questionnaire was derived from the literature on telemedicine satisfaction and experts in telemedicine [23-28]. We conducted a pilot test within our research group. The questionnaire consisted of three sections (Multimedia Appendix 1). The first part included demographic and clinical characteristics (age, gender, clinical specialty, etc). The second part consisted of statements that were rated on a 7-point Likert scale ranging from 1 (strongly disagree) to 7 (strongly agree). Statements were identified from previous literature that related to physicians’ perspectives on and attitudes toward telemedicine, such as overall satisfaction, behavioral intention, increasing the burden, safety issues regarding patient data, and hindering communication with patients, among others. In addition, we collected information about the current usage of telemedicine in their hospitals. The final section consisted of open-ended questions that included physician attitudes, concerns, and suggestions about telemedicine and any other comments related to telemedicine. The questionnaire was administered in a face-to-face manner.

### Data Gathering and Analysis

After completing the questionnaire, the data were tabulated and analyzed. All the physicians’ responses to the open-ended questions were entered into Microsoft Office Excel 2007 and were subjected to qualitative content analysis by reviewers. The analytical process was conducted by first cleaning the text, followed by extracting themes, and then developing categories. Free-text answers were summarized and assessed independently by two reviewers using a standardized evaluation process. A third reviewer reviewed by adjudication in cases of disagreement. The research team members repeatedly and independently read the answer summaries and validated the accuracy and meaning of the contents. Lastly, the results of the study were confirmed by all researchers in the team. The responses to the Likert scale–based statements were analyzed quantitatively by expressing them as whole numbers. The percentage of respondents who were in agreement with a statement was obtained by dividing the sum of the *strongly agree*, *agree*, and *somewhat agree* responses by the total number of responses to that statement. For questions using a 7-point Likert scale and questions that collected numerical demographic information, we reported mean values with standard deviations. For each clinical specialty, we calculated *P* values to determine the statistical significance of the differences between the scores of usability and willingness. Two-sided *P* values of .01 or less were deemed to meet statistical significance.

## Results

### Physician Demographics and Characteristics

We received 129 completed survey forms—direct survey handout and return on the day—with a response rate of 87.2% (129/148). Out of 129 respondents, 67 (51.9%) were females and 62 (48.1%) were males. The average age of the respondents was 35.6 (SD 3.9) years (range 23-48 years). The respondents came from 37 clinical specialties in 55 hospitals in China. These hospitals were located in 14 provinces (ie, municipalities) across China, including the three main provincial regions: Western China (n=5), Central China (n=4), and Eastern China (n=5). Among these 55 hospitals, 52 were 3A-level hospitals (ie, the highest level of hospital in China), two were 3B-level hospitals, and one was a 2A-level hospital. Table 1 shows the demographic characteristics of the respondents.

All hospitals in China are divided into three grades, each with three sublevels (ie, A, B, and C), with the highest grade being 3A. In principle, hospitals rated as a 3A-level hospital must meet very high standards in terms of beds, doctors, equipment, and quality of service.

**Table 1:** Demographic and clinical practice characteristics.

Participant demographics				Value (N=129)
**Age (years)**				
	Mean (SD)		35.6 (3.9)	
	**Range, n (%)**			
		23-29	4 (3.1)	
		30-39	105 (81.4)	
		40-48	20 (15.5)	
**Sex, n (%)**				
	Female		67 (51.9)	
	Male		62 (48.1)	
**Title, n (%)**				
	Resident		6 (4.7)	
	Senior physician		89 (69.0)	
	Specialist		34 (26.4)	
**Experience on the job** **(years)**				
	Mean (SD)		9.5 (4.5)	
	**Range, n (%)**			
		1-5	27 (20.9)	
		6-10	57 (44.2)	
		11-20	42 (32.6)	
		21-25	3 (2.3)	
**Electronic health record use (years)**				
	Mean (SD)		8.0 (2.8)	
	**Range, n (%)**			
		0-5	25 (19.3)	
		6-10	82 (63.6)	
		11-16	22 (17.1)	
**Provinces where hospitals were located per region (n=14), n (%)**				
	Western China^a^		5 (35.7)	
	Central China^b^		4 (28.6)	
	Eastern China^c^		5 (35.7)	
**Hospital level, n (%)**				
	3A		52 (94.6)	
	2A		2 (3.6)	
	3B		1 (1.8)	

^a^This includes Sichuan, Chongqing, Guangxi, Xinjiang, and Yunnan.

^b^This includes Shanxi, Henan, Hunan, and Jiangxi.

^c^This includes Beijing, Fujian, Guangdong, Shandong, and Liaoning.

### Current Use of Telemedicine

Among the 129 respondents, 94.6% (122/129) of the respondents’ hospitals adopted a telemedicine system. Only 5.4% (7/129) of the respondents did not know whether telemedicine was used in the hospital. A total of 34.1% (44/129) of physicians had never used a telemedicine system, 45.0% (58/129) used one occasionally (≤1 time/month), 11.6% (15/129) used one often (>1 time/month – <1 time/week), and only 9.3% (12/129) used one frequently (≥1 time/week). Depending on the question asked, 52% (44/85) of respondents were satisfied (responses of *strongly satisfied* plus *satisfied* and *somewhat satisfied*) with the telemedicine system (mean 4.7, SD 0.82).

Only 57 out of 129 (44.2%) physicians had participated in telemedicine training. A total of 32% (18/57) of those respondents were satisfied with their training (mean 4.2, SD 0.64). Among physicians who had used telemedicine systems, 11% (9/85) of them believed that electronic medical records were integrated into telemedicine. A total of 32% (27/85) of physicians believed that telemedicine had a decision support system (Table 2).

**Table 2:** Current use of telemedicine system.

Question or statement			Value (N=129)
Has your hospital adopted a telemedicine system? (yes), n (%)			122 (94.6)
**How often do you use the telemedicine system?, n (%)**			
	Not at all	44 (34.1)	
	≤1 time/month	58 (45.0)	
	>1 time/month – <1 time/week	15 (11.6)	
	≥1 time/week	12 (9.3)	
**What is your overall satisfaction with the telemedicine system?^a^ (n=85)**			
	Satisfied, n (%)	44 (51.8)	
	Score, mean (SD)	4.7 (0.82)	
	Score, range	3-7	
Have you taken telemedicine training? (yes), n (%)			57 (44.2)
**What is your overall satisfaction with the telemedicine training?^a^ (n=57)**			
	Satisfied, n (%)	18 (31.6)	
	Score, mean (SD)	4.2 (0.64)	
	Score, range	2-5	
**Does the telemedicine system integrate electronic medical records?^b^**			
	Yes, n (%)	9 (7.0)	
	Score, mean (SD)	4.2 (0.42)	
	Score, range	4-5	
**Does the telemedicine system integrate clinical decision support?^b^**			
	Yes, n (%)	27 (20.9)	
	Score, mean (SD)	4.3 (0.67)	
	Score, range	3-5	

^a^Satisfaction scores range from 1 (strongly dissatisfied) to 7 (strongly satisfied).

^b^Agreement scores range from 1 (strongly disagree) to 7 (strongly agree).

### Telemedicine During COVID-19

Of the 129 respondents, 60.5% (78/129) indicated that their specialty was suitable (responses of *strongly suitable* plus *suitable* and *somewhat suitable*) for adopting telemedicine during the COVID-19 pandemic (mean 5.0, SD 1.28). A total of 91.5% (118/129) of respondents would be willing to adopt telemedicine during the COVID-19 pandemic (mean 5.7, SD 1.02). In the group with telemedicine-appropriate specialties, obstetrics and gynecology had the highest mean value (mean 6.3, SD 0.97) and dermatology had the lowest mean value (mean 4.2, SD 0.75). Regarding willingness to adopt telemedicine, radiologists had the highest mean value (mean 6.4, SD 0.80) and ophthalmologists had the lowest mean value (mean 4.6, SD 0.49). For each specialty, we calculated *P* values to determine the statistical significance of the differences between the scores of usability and willingness (*P*>.01). The detailed attitudes and opinions about telemedicine on the part of the physicians are shown in Table 3.

**Table 3:** Physicians’ attitudes and opinions on the use of telemedicine in different subspecialties.

Specialty	Is telemedicine suitable for your specialty during the COVID-19 pandemic?^a^				Are you willing to use a telemedicine system during the COVID-19 pandemic?^b^				*P* value
	Score, range	Score, mean (SD)	Suitable (yes), n (%)	Score, range		Score, mean (SD)	Willing (yes), n (%)		
All (N=129)	2-7	5.0 (1.28)	78 (60.5)	3-7		5.7 (1.02)	118 (91.5)	N/A^c^	
Dermatology (n=5)	3-5	4.2 (0.75)	—^d^	5-7		6.2 (0.98)	—	.012	
Urology (n=6)	2-6	4.2 (1.21)	—	5-7		5.8 (0.90)	—	.03	
Laboratory (n=5)	3-7	4.2 (1.47)	—	4-7		6.0 (1.27)	—	.10	
Neurosurgery (n=6)	3-7	4.3 (1.25)	—	4-7		5.3 (0.94)	—	.18	
Nephrology (n=5)	4-5	4.4 (0.49)	—	5-7		6.0 (0.89)	—	.013	
General surgery (n=9)	2-7	4.6 (1.34)	—	4-7		5.4 (1.07)	—	.16	
Ophthalmology (n=5)	4-7	4.8 (0.75)	—	4-5		4.6 (0.49)	—	.67	
Pediatrics (n=9)	4-6	5.0 (0.67)	—	4-7		5.9 (1.10)	—	.07	
Anesthesiology (n=12)	2-7	5.1 (1.38)	—	5-7		6.0 (0.91)	—	.08	
Oncology (n=8)	4-7	5.3 (1.09)	—	5-7		5.6 (0.86)	—	.49	
Respiratory (n=6)	4-7	5.3 (0.94)	—	4-7		5.8 (1.07)	—	.45	
Cardiothoracic surgery (n=7)	4-7	5.5 (1.28)	—	5-7		6.1 (0.83)	—	.50	
Orthopedics (n=8)	3-7	5.8 (1.30)	—	5-7		6.0 (1.00)	—	.69	
Radiology (n=5)	5-7	6.0 (0.89)	—	5-7		6.4 (0.80)	—	.52	
Obstetrics and gynecology (n=8)	4-7	6.3 (0.97)	—	4-7		5.9 (1.05)	—	.50	

^a^This includes *strongly suitable* plus *somewhat suitable* and *suitable*. Suitability scores range from 1 (strongly unsuitable) to 7 (strongly suitable).

^b^This includes *strongly willing* plus *willing* and *somewhat willing*. Willingness scores range from 1 (strongly unwilling) to 7 (strongly willing).

^c^N/A: not applicable; *P* values were only calculated for individual specialties.

^d^The number of respondents who found telemedicine to be suitable and were willing to use it was not reported for individual specialties.

### Main Concerns of Adopting Telemedicine

Based on the findings of the survey, the major concerns regarding the use of telemedicine included the following: the inability to complete an in-person physical examination (101/129, 78.3%), the inability to communicate well with patients (32/129, 24.8%), the instability of the telemedicine system (30/129, 23.3%), and no assurance of patient medical safety (23/129, 17.8%) (Table 4).

**Table 4:** Major concerns regarding the use of telemedicine.

Major concerns	Respondents (N=129), n (%)
Cannot communicate well with patients	32 (24.8)
No assurance of patient medical safety	23 (17.8)
Inability to do an in-person physical examination	101 (78.3)
Unstable telemedicine system	30 (23.3)

### Barriers to the Use of Telemedicine

Overall, 58.9% (76/129) of respondents agreed that a physician’s inability to examine patients will hinder clinical decision making. A total of 44.2% (57/129) of respondents agreed that telemedicine makes it easier for patients’ data to be stolen, compromised, or hacked. Approximately one-quarter of the respondents (32/129, 24.8%) agreed that the lack of person-to-person contact in telemedicine can damage the doctor-patient relationship and trust. Only 15.5% (20/129) of respondents agreed that during the COVID-19 pandemic, the use of telemedicine will increase the burden on physicians (Table 5).

**Table 5:** Barriers to adopting telemedicine.

Barrier	Score, range	Score, mean (SD)	Respondents who agree^a^ (N=129), n (%)	Respondents who disagree^a^ (N=129), n (%)
The lack of person-to-person contact in telemedicine can damage the doctor-patient relationship and trust.	1-7	3.6 (1.89)	32 (24.8)	62 (48.1)
A physician’s inability to examine patients will hinder clinical decision making.	1-7	4.5 (1.02)	76 (58.9)	23 (17.8)
During the COVID-19 pandemic, the use of telemedicine will increase the burden on physicians.	1-6	3.0 (1.20)	20 (15.5)	87 (67.4)
Telemedicine makes it easier for patient data to be stolen, compromised, or hacked.	1-7	4.1 (1.23)	57 (44.2)	42 (32.6)

^a^Agreement includes *strongly agree* plus *somewhat agree* and *agree*. Disagreement includes *strongly disagree* plus *somewhat disagree* and *disagree.* Scores range from 1 (strongly disagree) to 7 (strongly agree).

### Physicians’ Comments

In the open-ended section of the questionnaire, a total of 127 respondents out of 129 (98.4%) made comments regarding the obstacles to adopting telemedicine and made suggestions for improving telemedicine (Tables 6 and 7). Two respondents did not make comments or suggestions about telemedicine.

The main barriers to implementation cited by physicians included the inability to examine patients personally (48/127, 37.8%), insufficient infrastructure support for telemedicine (40/127, 31.5%), issues concerning the quality of patients’ data (28/127, 22.1%), communication issues with patients (18/127, 14.2%), network issues (13/127, 10.2%), and lack of policy support (10/127, 7.9%). Table 6 lists the physicians’ comments regarding obstacles to the use of telemedicine.

Physicians believed that telemedicine could be promoted through the following incentives: performance measures (60/127, 47.2%), increased telemedicine equipment (22/127, 17.3%), policy support (21/127, 16.5%), financial support (19/127, 15.0%), technical support (18/127, 14.2%), increased training (18/127, 14.2%), and increased telemedicine publicity (14/127, 11.0%) (Table 7).

**Table 6:** Physicians’ comments regarding obstacles to the use of telemedicine.

Main obstacles to adoption of telemedicine^a^	Respondents (n=127), n (%)
Inability to examine patients personally	48 (37.8)
Insufficient infrastructure support for telemedicine	40 (31.5)
Issues concerning the quality of patients’ data	28 (22.1)
Communicating issues with patients	18 (14.2)
Network issues	13 (10.2)
Lack of policy support	10 (7.9)
Others^b^	49 (38.6)

^a^There were a total of 206 comments.

^b^Other comments included low patient acceptance (n=5), lack of funds (n=4), lack of performance measures (n=4), inadequate telemedicine promotion (n=3), etc.

**Table 7:** Physicians’ comments regarding promoting telemedicine.

Suggestions for promoting telemedicine^a^	Respondents (n=127), n (%)
Performance measures^b^	60 (47.2)
Increase telemedicine equipment	22 (17.3)
Policy support	21 (16.5)
Financial support	19 (15.0)
Technical support	18 (14.2)
Increase training	18 (14.2)
Increase telemedicine publicity	14 (11.0)
Others^c^	73 (57.5)

^a^There were a total of 242 comments.

^b^Performance measures included monetary incentives and professional incentives (eg, continuing education credits, facilitating physician promotions, and/or offering time-saving measures for physicians in other aspects of the workday).

^c^Other comments included developing guidelines for telemedicine (n=8), optimization of telemedicine systems (n=7), solving network issues (unable to connect, slow internet performance, etc) (n=5), including telemedicine coverage in health insurance (n=4), increasing the convenience of telemedicine (n=4), harmonious doctor-patient relationships (n=4), etc.

### Main Reasons for Being Willing or Unwilling to Use Telemedicine

Physicians’ attitudes toward telemedicine were positive, with 88.4% (114/129) of respondents stating that they were willing to adopt telemedicine. Only 8.5% (11/129) of respondents were unwilling to adopt telemedicine, and 4 respondents out of 129 (3.1%) were undecided about whether or not they were willing to adopt telemedicine. The main reasons physicians were willing to adopt telemedicine included convenience for patients (56/114, 49.1%), optimization of medical resources (31/114, 27.2%), and improving the level of medical care (16/114, 14.0%). The main reasons for being willing or unwilling to use telemedicine are given in Table 8.

**Table 8:** Physicians’ attitudes toward telemedicine.

Main reasons physicians were willing or unwilling to use telemedicine^a^			Respondents (N=129), n (%)
**Willing (n=114)**			114 (88.4)
	Convenient for patients	56 (49.1)	
	Optimized medical resources	31 (27.2)	
	Improved level of medical care	16 (14.0)	
	The trend of medical development	8 (7.0)	
	The COVID-19 pandemic	6 (5.3)	
	Others^b^	25 (21.9)	
**Unwilling (n=11)**			11 (8.5)
	The physician’s inability to personally examine a patient will hinder clinical decision making	6 (54.5)	
	More time spent	3 (27.3)	
	Low medical fees	2 (18.2)	
	Concerns about the quality of care	2 (18.2)	
	Cannot provide valid patient information	2 (18.2)	
	Others^c^	6 (54.5)	
Undecided			4 (3.1)

^a^There were a total of 163 reasons.

^b^Other reasons for being willing to use telemedicine included increased diagnosis and treatment efficiency (n=5), reduced patient burden (n=4), conducive to medical equity (n=2), reduced medical costs (n=1), enhanced patient satisfaction (n=1), etc.

^c^Other reasons for being unwilling to use telemedicine included low economic gain (n=1), patients’ distrust of telemedicine (n=1), medical malpractice (n=1), etc.

## Discussion

### Principal Findings

Although telemedicine has been used in various clinical specialties for decades [29], the emergence of the COVID-19 pandemic has highlighted the importance of telemedicine [30]. In the midst of the global COVID-19 catastrophe, a focus on telemedicine could play a critical role in the provision of global health care and may become a necessity for the general population [31]. In order to make the best use of telemedicine, we need to gain insight into physicians’ perceptions of telemedicine.

This study showed that the surveyed physicians had a high willingness to use telemedicine. The reasons for their high willingness were manifold but included the COVID-19 pandemic, telemedicine training courses, as well as young physicians in academic centers. The COVID-19 pandemic forced physicians to quickly adapt and use telemedicine [32]. Physicians’ willingness to adopt telemedicine may also be related to the COVID-19 pandemic’s movement-restriction policy [33]. Before answering the questionnaire, all the physicians spent more than 3 hours on coursework related to telemedicine. The telemedicine training course increased physicians’ awareness of, knowledge about, and attitudes toward telemedicine. There are studies that indicate that the knowledge and perception of health care professionals affect telemedicine adoption [34,35]. Moreover, younger physicians have a greater openness and willingness to adopt telemedicine [36]. One’s willingness to use telemedicine may also be influenced by one’s attitude toward telemedicine itself, one’s level of technology anxiety, and the patient-physician relationship [37]. These factors that were associated with a high willingness to use telemedicine were identified and must be considered in the long-term development of telemedicine.

Although telemedicine has found its way to nearly all clinical specialties, its use is uneven across specialties [38,39]. To promote the development of telemedicine in different specialties, we analyzed the willingness to use, and perceptions of, telemedicine on the part of physicians in different specialties. Due to the uneven distribution of the number of specialists, only specialties that included more than 5 participating physicians were analyzed. Although physicians’ willingness to participate in telemedicine was different from the usability of telemedicine in each specialty, there was no correlation between them.

The most obvious concerns and obstacles to telemedicine are limited in-person physical exams and the lack of vital sign assessment. The inability to complete an in-person physical examination was the highest concern for physicians (101/129, 78.3%) and was the main reason physicians cited it as a barrier to implementing telemedicine. This result is consistent with research from the United States [40]. This was mainly due to the concern by physicians that not being able to examine patients in person would affect clinical diagnosis. Whether in the learning stage or late in their careers, physicians want to carefully examine each patient personally. In telemedicine, the inability to examine the patient in person not only affects the physicians’ habits, but also sound and light present during telemedicine examinations can affect physicians’ diagnoses and treatment recommendations [41]. A well-lit environment and diffuse lighting to reduce glare allow physicians to detect physical examination findings more clearly, such as tremors, convulsions, and subtle facial expressions. Poor sound quality may limit understanding and mutual contact [41-44]. Therefore, health care professionals must be reassured that telemedicine is not a threat to their clinical decision making and that it could allow them to focus on patients who urgently need help. Some authors suggested that telemedicine might be best used in conjunction with face-to-face visits. Physicians can rely on proxies for examination [45].

An important aspect in the application of telemedicine will be the integration of telemedicine with the current health system workflows and the connection to the electronic health record [46]. In order to maximize the benefits of utilizing telemedicine technology, technologies including remote patient monitoring equipment need to be automatically synchronized to the patient’s chart, so that physicians can instantly obtain patient data [47]. Clinical decision support in telemedicine should also be enhanced to reduce medical errors.

This study suggests that there are many challenges and risks to telemedicine that need to be addressed before the technology is widely endorsed by physicians. These challenges may be due to regulation, incentives involving telemedicine, effective telemedicine training, malpractice insurance coverage for telemedicine, security and confidentiality of patient data, and telemedicine technology. These are in line with the findings of the other studies [48]. Physicians are less likely to use telemedicine if they are not adequately compensated for their time and effort [49]. Therefore, addressing the barriers to the development of telemedicine will require collaboration and efforts by health care institutions, policy makers, hospital administrators, physicians, and patients.

### Limitations of the Study

This study has potential limitations. First, this is a survey-based study and is subject to respondent bias inherent in all survey-based studies. Second, the survey was only about Chinese physicians. Incentive effects may differ in other countries due to cultural differences. Another limitation is the limited sample size and the descriptive nature of the study, which may not be able to reflect the opinions of all physicians in each hospital. However, considering the limited use of telemedicine in China and the lack of knowledge about telemedicine among general physicians, it is difficult to collect opinions through large random sampling. We recruited participants who were physicians and enrolled in a PhD program in clinical informatics. Most of them were also involved with the hospital management team. Therefore, in contrast to general physicians, they have a basic understanding of clinical informatics as well as medical information systems in their own hospital. In addition, the overall response rate was very high (87.2%) and included a variety of clinical specialties. The relatively younger physicians (23 to 48 years old) from the highest-level hospitals represented those who might be more familiar with telemedicine and digital technology. The responses were collected from 55 hospitals in Eastern, Central, and Western China, as it was a study representing various clinical subspecialties. Moreover, participants spent more than 3 hours on coursework related to telemedicine before completing the survey, so that they had a comprehensive understanding of telemedicine. The survey questions we asked were inherently pragmatic, and the responses to these questions faithfully reflected the physicians’ sentiments.

### Conclusions

The results of this survey indicate that, although telemedicine cannot yet be used universally for all health care needs and cannot fully replace in-person physical examinations, physicians’ willingness to use telemedicine was high. The modality of telemedicine is a tool worthy of careful evaluation and consideration by clinical subspecialties and their medical systems.

## Appendix

Multimedia Appendix 1 Telemedicine questionnaire.
