# Supplementary material for: Physicians’ Perspectives of Telemedicine During the COVID-19 Pandemic in China: Qualitative Survey Study
Source: JMIR Med Inform. 2021 Jun 1;9(6):e26463. doi: 10.2196/26463 (PMC8171288; doi:10.2196/26463)
Supplement: Multimedia Appendix 1 [file medinform_v9i6e26463_app1.pdf]

Appendix 1 Telemedicine Questionnaire

Gender: ☐Male ☐Female
Age: _____
Specialty: ______
Years of working experience: _________
The level of the professional role: ________
Years of using electronic health records: _________
Hospital: _________
City, Province: __________

1. Telemedicine is appropriate for your specialty during COVID-19.

1	2	3	4	5	6	7
Strongly Disagree	Disagree	Somewhat Disagree	Neutral	Somewhat Agree	Agree	Strongly Agree

2. You are willing to use telemedicine system during COVID-19.

1	2	3	4	5	6	7
Strongly Disagree	Disagree	Somewhat Disagree	Neutral	Somewhat Agree	Agree	Strongly Agree

1. The lack of person-to-person contact in telemedicine can damage the doctor-patient relationship and trust.

1	2	3	4	5	6	7
Strongly Disagree	Disagree	Somewhat Disagree	Neutral	Somewhat Agree	Agree	Strongly Agree

2. A physician's inability to examine patients will hinder clinical decision-making.

1	2	3	4	5	6	7
Strongly Disagree	Disagree	Somewhat Disagree	Neutral	Somewhat Agree	Agree	Strongly Agree

3. During COVID-19, the use of telemedicine will increase the burden on physicians.

1	2	3	4	5	6	7
Strongly Disagree	Disagree	Somewhat Disagree	Neutral	Somewhat Agree	Agree	Strongly Agree

4. Telemedicine makes it easier for patient data to be stolen, compromised, or hacked.

1	2	3	4	5	6	7
Strongly Disagree	Disagree	Somewhat Disagree	Neutral	Somewhat Agree	Agree	Strongly Agree

5. What are your major concerns regarding the use of telemedicine?
   a. Cannot communicate well with patients
   b. No assurance of patient medical safety
   c. Inability in-person physical examination
   d. Unstable telemedicine system

6. Does your hospital adopt telemedicine system?
   a. Yes b. No c. I don’t know

7. How often do you use telemedicine system?
   a. ≤1/Month b. ≤ 1/Week c. >1/ Week d. No

8. If you have used the telemedicine before, please select the overall satisfaction with telemedicine system.

1	2	3	4	5	6	7
Strongly Dissatisfied	Dissatisfied	Somewhat Dissatisfied	Neutral	Somewhat Satisfied	Satisfied	Strongly Satisfied

9. Have you taken any telemedicine training in your hospital?
   a. Yes b. No c. I don’t know

   If you choose a, please select the overall satisfaction with telemedicine training.

1	2	3	4	5	6	7
Strongly Dissatisfied	Dissatisfied	Somewhat Dissatisfied	Neutral	Somewhat Satisfied	Satisfied	Strongly Satisfied

10. Does the telemedicine system integrate electronic medical records?
    a. Yes b. No c. I don’t know

    If you choose a, please select the overall satisfaction with it.

1	2	3	4	5	6	7
Strongly Dissatisfied	Dissatisfied	Somewhat Dissatisfied	Neutral	Somewhat Satisfied	Satisfied	Strongly Satisfied

11. Does the telemedicine system provide any clinical decision support tools?
    a. Yes b. No c. I don’t know

    If you choose a, please select the overall satisfaction with it.

1	2	3	4	5	6	7
Strongly Dissatisfied	Dissatisfied	Somewhat Dissatisfied	Neutral	Somewhat Satisfied	Satisfied	Strongly Satisfied

12. What are your major concerns regarding the use of telemedicine?

____

____

____

____

____

13. Please provide some suggestions to promote the telemedicine.

14. Please list the main reasons why you are willing or unwilling to use telemedicine.
